# Suicide Literacy: A Call for National Training in Suicide Competencies for All Medical Doctors

**DOI:** 10.7759/cureus.93150

**Published:** 2025-09-24

**Authors:** Mona P Roshan, Sabrina Martinez, Kai Fu, Rodolfo Bonnin, Nathaly S Desmarais

**Affiliations:** 1 Department of Radiology, Florida International University Herbert Wertheim College of Medicine, Miami, USA; 2 Department of Psychiatry, Florida International University Herbert Wertheim College of Medicine, Miami, USA; 3 Department of Internal Medicine, Florida International University Herbert Wertheim College of Medicine, Miami, USA

**Keywords:** physician training, policy, prevention, public health, suicide

## Abstract

Suicide represents a critical global health crisis, with hundreds of thousands of lives lost each year. Despite its widespread impact, current practices for identifying and managing suicide risk in healthcare remain inconsistent and often inadequate. Primary care physicians are frequently the first and, sometimes, only point of contact for individuals experiencing suicidal thoughts. They also serve as the leading prescribers of mental health medications. However, formal training in suicide risk assessment and prevention remains highly variable, as standardized instruction has only recently gained national attention. At present, only a few states require suicide prevention training as part of physician licensure, underscoring a significant gap in preparedness. As suicide continues to rise as a leading cause of death, particularly among young adults, there is an urgent need to implement a national, structured training requirement. This narrative review draws upon published studies, national reports, and publicly available CDC data to highlight deficiencies in physician training and advocate for a unified, evidence-based approach to ensure that healthcare providers are equipped to recognize and respond to suicide risk effectively. We conclude that integrating mandatory, structured suicide prevention training into physician licensure requirements is a necessary step to enhance clinical preparedness, improve patient outcomes, and contribute to reducing suicide rates nationwide.

## Introduction and background

Suicide is a global challenge, claiming over 800,000 lives annually [1-4]. In the United States, it ranks as the 11th leading cause of death, with nearly 50,000 lives lost in 2023; firearms accounted for 55.3% of cases [5]. It is the second and third leading cause of death among those aged 15-34 [5]. In 2021, 12.3 million adults reported suicidal ideation, 3.5 million made plans, 1.7 million attempted suicide, and 48,183 died by suicide [5]. From 1999 to 2018, the US age-adjusted suicide rate rose 35% (10.5-14.2 per 100,000), contrasting with declines in other leading causes of death [2,3]. While this paper focuses on the US, similar gaps in physician preparedness are seen internationally, underscoring the global relevance of this issue.

Over 75% of individuals who died by suicide had seen a healthcare provider in the previous year, and 44% had seen a primary care provider within the last month [6-8]. These findings highlight the need for all physicians to acquire skills in suicide risk assessment and prevention. The role of primary care physicians (PCPs) is especially critical, as they prescribe more than 75% of antidepressants and are often the first point of contact for patients with mental health needs [9].

In 2021, about one in five US adults (50 million) lived with a mental illness [10]. Yet, many PCPs report discomfort treating psychiatric conditions [11]. The American Academy of Family Physicians recommends curricular guidelines in human behavior and mental health, including screening, pharmacology, and motivational interviewing [12], but PCPs still report a lack of confidence [11].

Psychiatrists, who make up only 4% of the physician workforce, face overwhelming demand, with caseloads averaging 8,400 individuals compared to 2,714 per PCP [13]. This shortage leads to long waits, 67 days for in-person and 43 days for telehealth visits [14], and many psychiatrists no longer accept insurance, creating financial barriers [15]. Patients presenting to emergency rooms with suicidal ideation also face high suicide and mortality risk in the year following discharge [16], revealing a gap in continuity of care. Even brief evidence-based interventions in healthcare settings reduce future suicide attempts and increase follow-up with mental health providers [17]. These findings support integrating comprehensive suicide risk training into physician licensure requirements.

Because physicians frequently encounter patients at risk, they are well positioned to conduct suicide assessments and provide targeted interventions. Prior reviews of US suicide training policies have also called for expanded training for PCPs and mental health professionals [10,18]. The 2024 National Strategy for Suicide Prevention emphasized the need to integrate suicide assessments across healthcare, specifically highlighting PCPs [19], and urged policy changes to require training for licensure [19]. Experts consistently stress that adequately trained healthcare professionals play a pivotal role in reducing suicide [3,18-20].

## Review

Suicide training requirements

To maintain continuity in state medical licensure, physicians must complete continuing medical education (CME) training to maintain clinical competencies within the medical field. CME activities are intended to maintain and increase the knowledge and skills that physicians use to provide services for patients, the public, and the profession. Additionally, physicians are required to take CMEs to keep their hospital and medical board privileges.

Requiring education on specific topics within CME requirements is not a new phenomenon. States can mandate training on certain issues based on persistent and growing societal concerns, one such example being opioid prescription education to combat the opioid epidemic, which is now required in 47 states [21]. Suicide accounted for nearly 50,000 deaths in 2021 in the United States, while opioid overdose deaths totaled over 75,000 that same year, the vast majority of which were due to illicit synthetic opioids, rather than prescription opioids [5,22]. Nonetheless, far more states have mandatory CME training requirements on opioid prescribing than on suicide prevention (CME requirements). Only three states currently require physicians to receive suicide assessment training, and even among those, there is no continuity in the requirements (Table 1).

**Table 1:** Differences between states requiring suicide training State-specific suicide prevention training requirements for physicians are based on publicly available information from the Utah Department of Commerce [23], the Washington State Department of Health [24], and the Nevada State Board of Medical Examiners [25]. CME, continuing medical education

State	Requirement
Nevada	Two hours of training within two years of initial licensure, then two hours every four years thereafter
Utah	Minimum of one online suicide prevention course, from a list provided by the Utah Department of Commerce Division of Occupational and Professional Licensing
Washington	One-time six-hour CME on suicide prevention during the first full CME reporting period

**Figure 1:**
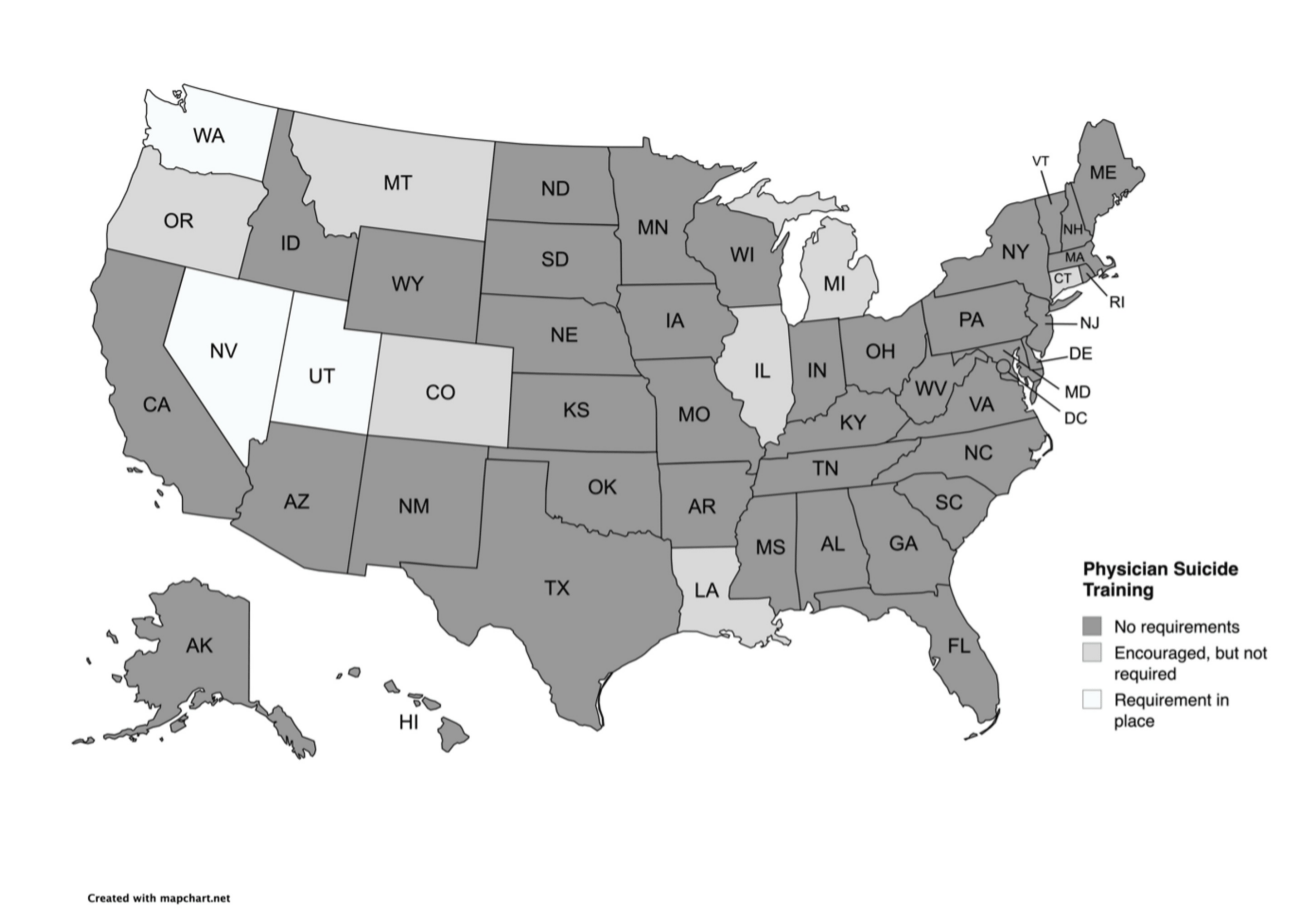
Suicide training requirements for physicians by state States are categorized based on whether physician suicide prevention training is required, encouraged, or not required. This figure was created using MapChart.net. Image credit: Sabrina Martinez

**Figure 2:**
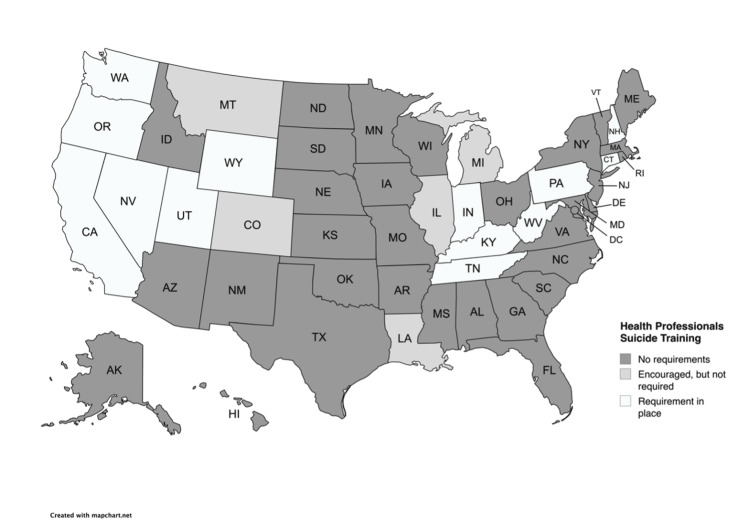
Suicide training requirements for mental health professionals by state States are categorized based on whether suicide prevention training for mental health professionals is required, encouraged, or not required. This figure was created using MapChart.net. Image credit: Sabrina Martinez

The three states that require physicians to complete CME training on suicide prevention are Nevada, Utah, and Washington (Figure 1). Since March 2012, the state of Utah has required physicians to complete an online suicide prevention training course every two years, selected from a list provided by the Utah Department of Commerce (Table 1) [23]. Since January 2016, the state of Washington has required physicians to take a one-time six-hour course on suicide assessment, treatment, and management (Table 1) [24]. Since July 2017, the state of Nevada has required physicians to complete at least two hours of training on suicide detection, intervention, and prevention within two years of initial licensure and every four years thereafter (Table 1) [25]. Seven states (Colorado, Hawaii, Illinois, Indiana, Louisiana, Michigan, and Montana) have recommendations for physicians to complete suicide prevention training, but no mandatory requirement (Figure 1) [18]. For example, to further promote the completion of these trainings, Montana has a state suicide prevention coordinator, Karl Rosston, LCSW, who oversees a training program for medical professionals on recognizing the early warning signs of suicidality, depression, and other mental illnesses [18]. The suicide prevention coordinator works in the Montana Department of Public Health and Human Services and is generally responsible for increasing public awareness, maintaining training materials, allocating funding for training and interventions, and creating and regularly updating a state suicide reduction plan. The remaining 40 states do not require suicide prevention training for physicians renewing their licenses. This contrasts with other mental health professions that have state-mandated training requirements. For example, at least nine states mandate training in suicide assessment, treatment, and management for mental health professionals (Figure 2). These states include California, Indiana, Kentucky, Nevada, New Hampshire, Pennsylvania, Tennessee, Utah, and Washington (Figure 2) [18].

Using publicly available CDC data from 2017 to 2021, we conducted a descriptive analysis comparing suicide rates in states with and without CME requirements for suicide prevention training [5]. The three states with such mandates experienced a modest decline in average suicide rates, from 19.96 to 18.96 per 100,000, while states without these requirements showed relatively stable rates, ranging from 16.49 to 16.84 per 100,000. Although these trends may suggest a potential association, the findings should be interpreted with caution. The small number of states with mandates, variation in policy content and implementation, and multiple confounding factors, including differences in mental health funding, social determinants, and broader public health strategies, limit the ability to draw causal conclusions. The medical literature underscores that suicide rates are shaped by a complex interplay of social, economic, and healthcare system variables, and that state-level comparisons are particularly vulnerable to ecological bias.

Despite this observed trend, reliable and objective methods to evaluate the direct impact of mandated physician training on suicide rates remain limited. Observed changes may be confounded by a variety of factors, including differences in state-level investment in mental health and suicide prevention programs. Future research may benefit from more granular analyses, such as comparing suicide outcomes among patients treated by physicians in states with versus without mandated suicide prevention training.

In addition, psychiatrists have reported being undertrained during residency [26]. Although over 90% of residency programs hold formal educational sessions regarding suicidal patients, the topic of how to deal with suicidal patients accounts for less than four lectures across the entire residency training program, on average [26]. While more than 90% of residencies reported offering training opportunities for residents to perform suicide risk assessments, only 27.5% of residencies held workshops to actively develop residents’ skills, while the remaining portion provided passive training techniques [27].

Impact of training on physician competency

Medical students, residents, and physicians often report feelings of discomfort when dealing with suicidal patients. Many studies revealed that most medical students, regardless of location and nationality, carry negative attitudes toward suicidal patients [14,28]. More specifically, most believe that suicidal patients are lonely and depressed and believe that suicidal behavior stems from traits associated with weak personality and self-destructive tendencies [28]. In another study, about 40% of pediatric residents indicated they were uncomfortable conducting patient suicide risk assessments [29], despite about 75% of respondents reporting seeing suicidal patients on a regular basis in their primary care practice. Another qualitative study of young physicians’ experiences highlighted challenges in establishing connections, maintaining competence, and managing emotional reactions when dealing with suicidal patients [30]. They reported emotional distress, grappling with practical concerns, fears of making incorrect decisions, and the weight of responsibility for the patient’s life, while also citing drawing on personal experiences to inform their approach.

Research consistently demonstrates that training significantly boosts doctors’ confidence and ease in addressing suicide prevention. For instance, a study involving over 2000 healthcare professionals revealed a notable increase in their assurance in managing suicide risks following training sessions [1]. Similarly, an evaluation of a training program including 873 healthcare professionals in Washington state showed enhanced knowledge and attitudes toward suicide prevention [31]. Notably, participants reported a substantial reduction in discomfort when discussing strategies like safe firearms and medication storage. Moreover, insights from the Zero Suicide Workforce Survey underscore the efficacy of even brief training sessions in augmenting healthcare professionals' skills and confidence in suicide prevention [4]. Furthermore, a study of 500 primary care providers in Stockholm demonstrated that after receiving adequate training, they experienced heightened clarity, job confidence, and a positive outlook regarding their effectiveness in preventing suicide [32]. Similarly, the OSPI-Europe study highlights the effectiveness of standardized training programs in enhancing general practitioners’ knowledge, attitudes, and confidence in managing depression and suicidal ideation [33]. Despite baseline stigmatizing attitudes and low confidence levels, the training led to significant improvements in these areas, emphasizing the importance of suicide prevention efforts within primary care settings. Standardized training programs play a crucial role in enhancing healthcare professionals’ capacity to effectively address suicide prevention in primary care settings by boosting confidence, knowledge, and attitudes.

Impact of suicide training programs

Increasing the confidence of healthcare providers, including physicians, in their ability to treat suicidal patients is imperative due to the suggested association between lower confidence and hindered management of patients with suicidal thoughts [1]. Increased knowledge regarding suicide has been significantly correlated with higher confidence in the skills required when working with suicidal patients [4,34]. The “All Patients Safe Program,” developed in response to a large increase in individuals who required suicide training, resulted in a significant increase in the knowledge, attitude, and confidence of participants in dealing with patients with suicidal thoughts, 53% of whom were physicians [31]. Suicide training programs across Europe have also been shown to improve the outlook, understanding, and confidence of providers in suicide prevention in multiple studies [1,33]. Practitioners’ confidence, specifically, has been shown to significantly increase for an extended period of time following suicide training [1,33]. Additionally, longer training programs and those that require an increased level of active participation also resulted in higher suicide skills confidence [4,34].

Although implementing training models for PCPs has consistently resulted in increased confidence in physicians when dealing with this population, some studies have not shown a significant decrease in rates of suicide after the implementation of a suicide training model [1,31]. However, other studies have shown a reduction in suicide rates [1]. The literature surrounding the effects of suicide training is conflicting, both bringing light to the complexity surrounding risk factors for suicide as well as the necessity of implementing and studying the effects of more structured programs. Notably, a 10-year systematic review of the literature found that one of the most effective methods to reduce suicide rates was training PCPs in depression recognition and treatment strategies [20]. A second systematic review also found that education of PCPs significantly decreased rates of suicide and even reduced suicidal ideation [3]. Additionally, the systematic review showed that repetitive training sessions show a continued reduction in suicide rates over the following years when compared to a one-time training session, supporting the idea that suicide prevention training should not be a one-time session but rather should be required at regular intervals [3].

Impact of training on patient care

Trust within the doctor-patient relationship is always important, but this trust becomes essential in the areas of mental health and suicidality. Patient trust increases the likelihood of patients disclosing suicidal ideation, which is crucial for timely intervention and effective suicide prevention. Research has demonstrated that programs like Mental Health First Aid (MHFA), Applied Suicide Intervention Skills Training, and the Zero Suicide framework help to equip providers with the tools to recognize warning signs and engage in meaningful conversations with at-risk patients, empowering providers to have the knowledge and comfort needed to help patients who have disclosed suicidal ideation. A meta-analysis of MHFA training programs showed improvements in mental health knowledge, reductions in stigmatizing attitudes, and increases in helping behaviors, underscoring the effectiveness of these trainings [35].

There is a need for suicide prevention initiatives within primary care settings, specifically, given that primary care serves as the primary point of contact for suicidal patients within the healthcare system. It is imperative to prioritize integration between medical and behavioral health services for effective suicide prevention. Training programs that emphasize this integration can significantly improve patient outcomes. Research indicates that integrated care models, which combine medical and behavioral health services, lead to better identification and management of individuals at risk of suicide [7]. Programs like Collaborative Care, Integrated Behavioral Health Training, and the Zero Suicide initiative help providers develop the skills and confidence necessary to offer comprehensive, evidence-based care, addressing both physical and mental health needs [2,36].

Implementing the Zero Suicide framework has led to substantial reductions in suicide rates among patients, with some health systems reporting decreases of up to 75% [2,37,38]. These reductions underscore the vital role of these training programs in primary care settings. Moreover, the integration of such training improves overall patient care by fostering a holistic approach that considers both mental and physical health. Patients benefit from a more supportive and responsive healthcare environment, leading to better mental health outcomes, increased satisfaction with care, and reduced feelings of hopelessness and distress [39,40]. By fostering a trust-based relationship and incorporating a variety of training approaches, primary care providers can play a pivotal role in reducing suicide rates and improving overall patient care and mental health status.

Discussion

Despite suicide being a major public health issue, claiming over 800,000 lives worldwide yearly, many physicians lack the skills and confidence needed to assess suicidal patients and implement effective interventions. Most patients who died by suicide visited their PCP in the year before their death, and almost half had visited their PCP in the month before their death, placing physicians in a unique position to assess and treat suicidal patients.

In a landscape where psychiatrists are in high demand and relatively low supply, PCPs often are the first and most consistent point of contact for patients with mental health concerns. PCPs prescribe a significant percentage of anti-anxiety and antidepressant medications, yet their training in suicide prevention varies widely. This inconsistency results in significant differences in their preparedness to handle mental health crises, highlighting a systemic failure to equip them with the necessary skills to address and manage suicidal patients. Many studies have revealed that physicians feel uncomfortable and ill-equipped to manage suicidal patients. Despite frequently seeing patients at risk for suicide and the subsequent need for PCPs to receive standardized training, only three states mandate suicide prevention training for all physicians, and seven additional states recommend training.

Multiple studies have indicated that targeted training significantly increases the levels of confidence and knowledge of providers in assessing and treating patients at risk of suicide [1,4,31,33]. These studies also noted improvements in attitudes toward these patients. Physician confidence, in particular, has been shown to significantly increase for a long period after training, which is important due to the association between provider confidence and the ability to manage patients with suicidal thoughts. Although research has been mixed, the strongest and most compelling research on methods to reduce suicide rates cited that training PCPs on suicide management is one of the most effective methods of reducing suicide rates [3,20]. It is important to note that studies further show that longer, consistent, and active training results in increased levels of confidence in suicide skills training [4,34]. This same trend has been found in the decrease in suicide rates, where consistent training results in a continued reduction in suicide rates in the following years [3].

Thus, research shows that consistent and active suicide prevention training helps to increase confidence and knowledge of physicians, which in turn leads to more evidence-based and effective care for patients and significantly decreased suicide rates in affected populations. By mandating that licensure requirements include structured training programs in suicide assessment and prevention for all physicians, we can significantly enhance their competencies and further impact outcomes on suicide rates.

Limitations

This paper acknowledges several limitations inherent in the analysis of suicide prevention training for healthcare professionals across all states. Suicide has a long history of stigma within our society, and no information was provided about why seven states (Colorado, Hawaii, Illinois, Indiana, Louisiana, Michigan, and Montana) have training recommendations and why three states (Nevada, Utah, and Washington) have training requirements. The long-term sustainability of training effects remains uncertain, requiring longitudinal studies to assess the durability of training interventions and their impact on suicide prevention efforts over time.

Despite these limitations, efforts to enhance suicide prevention training represent a critical step toward reducing the burden of suicide and improving mental health outcomes. Continued research, evaluation, and collaboration are essential to address these limitations and advance our understanding of effective strategies for suicide prevention.

## Conclusions

Forty-four percent of patients had visited their primary care provider in the month before dying from suicide. Physician competencies that address suicide assessment and intervention cannot be ignored. Due to the critical role and likelihood of contact between physicians and persons at risk of suicide, integrating comprehensive suicide prevention training as part of licensure requirements for all physicians is essential to increase skills in literacy, confidence, and comfort levels. This will, in effect, improve consistency and quality in care across the medical community and make a strong contribution to the national suicide prevention efforts, as well as a commitment to prioritizing mental health and saving lives.
